# COSMIN Review About Assessment Tools for Sexuality Knowledge in People with Intellectual Disability

**DOI:** 10.1007/s10508-025-03260-w

**Published:** 2025-11-24

**Authors:** Verónica Estruch-García, María Dolores Gil-Llario, Olga Fernández-García, Vicente Morell-Mengual, Rafael Ballester-Arnal

**Affiliations:** 1 Department of Developmental and Educational Psychology, Faculty of Psychology, University of Valencia, Av. Blasco Ibáñez, 21, 46010 Valencia, Spain; 2 Department of Basic and Clinical Psychology and Psychobiology, Faculty of Health Sciences, Jaume I University, Castellón de la Plana, Spain

**Keywords:** Sexual knowledge, Intellectual disability, Psychometric properties, COSMIN, DSM-5-TR

## Abstract

Despite the relevance of assessing the level of sexuality knowledge in people with intellectual disability, appropriate tools for measuring this domain are scarce. Our aim was to critically appraise, compare, and summarize the measurement quality and psychometric properties of all self-reported sexuality knowledge questionnaires for people with intellectual disability. To this end, a systematic search of the Scopus, Web of Science, PubMed, EMBASE, and MEDLINE databases was performed. This review was based on the COSMIN methodology for systematic reviews of patient‐reported outcome measures. Eighteen studies were found that reported on the measurement properties of nine assessment tools. The psychometric properties of the instruments were generally indeterminate or not reported due to incomplete data, and the use of statistical methods not preferred according to the criteria of good psychometric measures. There was moderate quality evidence of the psychometric properties of the scales. The Detection of Sexual Abuse Risk Screening Scale and the Inventory of Sexual Knowledge of People with Intellectual Disability are instruments that have demonstrated potential for use, but their psychometric quality for non-Spanish populations should be assessed. In conclusion, future studies on property measurement instruments for the assessment of sexual knowledge are needed. Therefore, the instruments should be updated because most studies were performed nearly a decade ago.

**Supplementary Information:**

The online version contains supplementary material available at 10.1007/s10508-025-03260-w.

## Introduction

Sexual health is a human right and an essential part of overall well-being throughout the life span. It is not merely the absence of disease or abuse, but the ability to enjoy safe, pleasurable, and consensual sexual experiences, supported by access to information, services, and respect for one’s autonomy and dignity (World Health Organization, 2006). However, the sexual health of people with intellectual disabilities remains largely overlooked, often due to social discomfort and persistent stereotypes that portray them as asexual, hypersexual, or incapable of making informed choices about their bodies and relationships (Darragh et al., 2017). Such misconceptions frequently result in the denial of comprehensive sex education, in both family and institutional contexts (Borawska-Charko et al., 2017), leaving this population without the necessary tools to understand and navigate their sexuality, understood not only as sex and reproduction, but also as gender identity, sexual orientation, intimacy, eroticism, and emotional connection (World Health Organization, 2006).

Research consistently shows that many individuals with intellectual disabilities have limited sexual knowledge, especially regarding reproductive health, consent, and disease prevention (Jahoda & Pownall, 2014). Leutar and Mihoković (2007), for instance, found striking misconceptions, such as the belief that pregnancy can result from kissing or eating watermelon, illustrating the urgent need for accurate, accessible sexual education. These knowledge gaps can compromise their ability to make informed decisions, increasing their vulnerability to misinformation, exploitation, and adverse sexual health outcomes.

Moreover, insufficient knowledge may increase individuals’ vulnerability to sexual abuse, particularly when the perpetrators are relatives (Martinello, 2014; Mitra et al., 2016). In fact, the prevalence of sexual abuse among people with intellectual disabilities is higher than that in the general population (Tomsa et al., 2021).

Despite these findings, other researchers have reported varied levels of knowledge (Gil-Llario et al., 2020; Schaafsma et al., 2017), suggesting inconsistencies that may stem from the different methodologies used to assess sexual knowledge. Some studies on the level of knowledge have used semistructured interviews (Darragh et al., 2017; Leutar & Mihoković, 2007; Long et al., 2011), while others have used self-report questionnaires. This latter type of instrument is the most commonly used (Gil-Llario et al., 2021; Liou, 2014, 2022; McCabe et al., 1999; Talbot & Langdon, 2006). However, many of these tools were developed decades ago, so some aspects of modern sexual issues, such as sexting and online relationships, were not integrated (Thompson et al., 2016).

Furthermore, these instruments are necessary because they allow us to design sexual education programs based on what they know and what they need to learn, evaluate the effectiveness of sexual education programs, and compare their results (Liou, 2022). However, Estruch-García et al. (2021) discovered that many studies adapt questionnaires initially intended for individuals without intellectual disability (Hayashi et al., 2011; Navarro et al., 2010) or use specially developed tools whose reliability remains unexamined (Chodan et al., 2017; Gutiérrez-Bermejo et al., 2021; van den Toren et al., 2021; Vizcaino & Aciego, 2015; Yueh-Ching et al., 2020), leading to less reliable findings (Grieveo et al., 2006). However, some researchers have used tools with proven psychometric properties. For example, the effectiveness of the Saludiversex Program has been evaluated (Gil-Llario et al., 2023a) with ISK-ID (Gil-Llario et al., 2021) and DSARs (Gil-Llario et al., 2020).

Creating assessment tools tailored to this population faces additional challenges due to their specific learning needs, which may affect the reliability and validity of these instruments. Finlay and Lyons (2001) noted that people with intellectual disability might exhibit response biases, such as a tendency to affirmatively answer questions, regardless of their content, and are more susceptible to suggestive questioning. They also mentioned studies such as Sigelman et al. (1982) and Malik et al. (1991), who found that yes–no questions yield greater responsiveness and reliability than other types of questions, such as open-ended questions or multiple-choice questions. However, Hartley and MacLean (2006) concluded that the responsiveness, reliability, and validity of Likert-type scales in people with intellectual disability are comparable to those of yes/no scales, either/or open-ended questions. They also suggested that Likert-type scales might be more suited for people with borderline or mild intellectual disability than for those with moderate to profound intellectual disability, especially when pictorial representations of options are not employed.

Therefore, this study aimed to critically appraise, compare, and summarize the measurement quality and psychometric properties of self-reported sexuality knowledge questionnaires for people with intellectual disability.

## Method

A systematic review was performed because it allows us to critically appraise, compare, and summarize the measurement quality and psychometrical properties of all self-reported sexuality knowledge questionnaires for people with intellectual disability. This review adhered to the COSMIN methodology for systematic reviews of Patient‐Reported Outcome Measures (PROMs), following the Cosmin Guidelines for Systematic Reviews of Patient-Reported Outcome Measures (Prinsen et al., 2018), to ensure the methodological rigor of the research.

### The Eligibility Criteria

The inclusion criteria were as follows: (1) the aim of the PROM is to measure the level of knowledge about sexuality; (2) the sample consists of people with intellectual disability; (3) the aim of the study is the assessment of the measured properties, the development of a PROM (to rate the content validity), or the evaluation of the interpretability of the instrument; and (4) publications up to February 2023.

The exclusion criteria were as follows: (1) studies in which the PROM was used to measure outcomes (e.g., in randomized controlled trials); (2) studies in which the PROM was used in a validation study of another instrument; (3) studies in which other-reported measurements were used; (4) books and book chapters, guides, theses, conferences, and press articles due to the difficulty in retrieving these documents; (5) studies that involved interviews; (6) reviews; and (7) studies in which tools were used to assess other constructs.

### Search Strategy

A systematic search was conducted in five electronic databases: Scopus, Web of Science, PubMed, EMBASE, and MEDLINE. The search strategy was based on analyzing PROM(s) that measure the level of sexual knowledge of people with intellectual disability, incorporating terms such as “disorder intellectual development” or “intellectual functional diversity.” These terms were included because they have been consistently used in prior versions of the DSM-5-TR (American Psychiatric Association, 2022). It was combined with keywords used to refer to sexual knowledge such as ‘knowledge’ or ‘understanding’ AND ‘sexuality’ or ‘sexual health’ and descriptors such as ‘measure’, assessment’ or ‘scale’. These were inputted using Boolean operators (see Table 1). The complete search strategy for each database is detailed in Appendix 1. The results from the ProQuest Dissertation & Thesis database were refined by document type (article) and area of study (limited to Psychology, Medicine, Social Sciences, Health Professions, and Nursing) because of the high number of unrelated studies previously found. Language restrictions were not applied.

**Table 1:** Search strategy

Construct	Population	Type of instruments	Measurement properties
(Knowledge OR understanding) AND (Sexuality OR ‘sexual health’ OR ‘sexual abuse’ OR ‘sexual relationships’ OR violence OR ‘gender identities’ OR ‘gender roles’ OR anatomy OR ‘intimate parts’ OR ‘sexual orientation’ OR eroticism OR pleasure OR intimacy OR reproduction OR ‘sexually transmitted diseases’ OR ‘sexual risk behaviour’)	(‘intellectual disability’ OR ‘disorder intellectual development’ OR ‘intellectual functional diversity’ OR ‘mental retardation’)	(measure* OR assess* OR tool OR instrument OR scale OR question* OR battery OR inventory OR index OR form OR evaluation)	(Construction OR psychometric properties OR develop* OR validity OR validation OR reliability OR responsiveness OR interpretability)

### Data Extraction

First and last name initials are used to describe the author’s role in the systematic review. The keyword combination was introduced in the databases by the researcher VMM. After removing duplicates, MDGL and RBA screened the remaining titles and abstracts using the eligibility criteria. The selected articles were independently and thoroughly read and analyzed by both authors, who applied the inclusion criteria. For data management and analysis in this systematic review, RevMan (Review Manager) was used. Disagreements were reviewed by a third author (VMM). For each selected article, the data were extracted by two of the authors (RBA and MDGL). The data included details of the study characteristics (e.g., study purpose, assessed psychometric properties, characteristics of the sample); instrument details (e.g., instrument names, construct to be measured, target population, purpose of use, number of [sub] scales and items, response options, and recall period); and study results on seven psychometric properties (internal consistency, reliability, measurement error, structural validity, hypothesis testing, cross-cultural validity, and criterion validity).

### Evaluation of the Methodological Quality of the Studies

The methodological quality of the studies was assessed using the COSMIN Risk of Bias checklist (Mokkink et al., 2018; Terwee et al., 2018). This systematic approach ensured a comprehensive assessment across multiple domains, such as content validity, structural validity, internal consistency, cross-cultural validity, measurement invariance, reliability, measurement error, criterion validity, construct validity, and responsiveness. This checklist contains 10 boxes, which have 3 to 35 items each. Each standard in a box is scored on a four‐point scale (“very good,” “adequate,” “doubtful,” and “inadequate”). The response option “not applicable” is available for some standards. The methodological quality score for each psychometric property was obtained by the “worst score” method, as recommended by the COSMIN guidelines (Mokkink et al., 2018). Therefore, the lowest rating of any of the items in a psychometric property checklist is taken as the overall score for that property. Then, the psychometric property of each study was scored against the criteria for good measurement properties (Prinsen et al., 2018; Terwee et al., 2018). Scores were defined as a positive rating “+,” indeterminate rating “?,” or negative rating “-.” Finally, the quality of the evidence was graded by using the GRADE approach. Most ratings were independently assessed by two reviewers (VEG and OFG). The instruments included in this review that had been developed and validated by one or more of the undersigned authors were evaluated by those coauthors who had not participated in the development and validation process. Nonconsensual ratings were determined by discussion between reviewers (see Tables 2, 3, 4, 5).

**Table 2:** Characteristic of the included instruments for the assessment of sexual knowledge for people with intellectual disabilities

PROM (ref)	Construct	Purpose of use and target population	Total items	(Sub)scales and items	Response option	Range of scores
BSK. Bender Sexual Knowledge Questionnaire (Bender et al., 1983)	Sexual Knowledge	Assessing students’ initial level of knowledge and the change in their amount of knowledge in the various areas being taught in a course on human relations and sex education in people with mentally handicapped	61	Physiology: 14; Pregnancy: 7; Sex act: 13; Masturbation: 9; Contraception: 10; Types of sexuality: 4; Venereal disease: 4	Each correct answer receives 1 point	Total: 0–69 Subscales: Vary from 0–16 to 0–10
ASK. Assessment of Sexual Knowledge (Butler et al., 2003; Galea et al., 2004)	Sexual knowledge	Assessing the sexual knowledge of adults with intellectual disability	KS: 124 KQ: 25	Parts of the Body: 20; Public and Private: 6; Puberty: 6; Menstruation: 5; Menopause: 2 Masturbation: 5; Relationships:11 Protective Behaviours:7; Sexuality: 13; Safer Sex Practices: 2; Contraception: 15; Pregnancy & Birth: 4; Sexual Health-Screening Tests: 8; Sexually Transmitted Infections: 6; Legal Issues Regarding Sexuality: 14	KS: NR QKQ: Dichotomous scale (“*Yes”* or “*No”*) Each correct response count as “1” and each incorrect response count as “0.” Some items require more than one response in order to receive a score of 2	KS: 0–248 QKQ: 0–25
DSARss. Detection of Sexual Abuse Risk Screening Scale (Gil-Llario et al., 2020)	Sexual Abuse Risk	(a) Assessing of sexual abuse risk in adults with mild and moderate ID, (b) be equally applicable to both men and women, (c) identify different vulnerability domains as well as a general index of SA risk, (d) accurately predict the risk of being a victim of different forms of SA (unwanted sexual touching, SA, and coercion, etc.)	19	Acceptance of the abuse due to affection: 4; Denial of the risk associated with places: 3; Risk factors and self-protection skills: 8; Lack of awareness of intimacy rules: 4	Dichotomous scale (“*True* “or “*False”)*	0–19
ISK-ID. Inventory of Sexual Knowledge of People with Intellectual Disability (Gil-Llario et al., 2021)	Sexual knowledge	(a) Assessing sexual knowledge in adults with mild intellectual disability; (b) Identifying the level of knowledge in different domains, as well as a general index of sexual knowledge; and (c) monitoring changes in sexual knowledge because of the implementation of sexual education programs	34	Concept of sexuality: 4; Body image and sexual communication: 5; Sexual practices: 7; Homosexuality: 3; Dating, intimacy and assertiveness: 8; Sexual health: 7	Dichotomous scale (“*Yes”* or “*No”*)	0–34
Illustrated Scale Measuring the Sexual‐Abuse Prevention Knowledge of Female (Liou, 2014)	Sexual‐Abuse Prevention Knowledge	Measuring the sexual-abuse prevention knowledge of female students with intellectual disabilities studying at special education schools	30	Puberty physiology: 6; Body boundaries: 6; Identification of improper sexual relationships: 6; Identification of abusive situations: 6; Coping methods when facing abusive situations:6	Dichotomous scale (“*True* “or “*False”)* for 20 items and 10 multiple-choices Each correct response count as “1” and each incorrect response count as “0.”	0–30
Pictorial Sexual Knowledge Scale for Male (Liou, 2022)	Sexual knowledge	Measuring the sexual knowledge of male students with Intellectual Disabilities studying at special education schools	26	Puberty and Physiology: 4; Body Boundaries: 5; Proper Touching: 4 Proper Relationships: 4; Sexually Transmitted Diseases (STD): 4; Sex and the Law: 5	Multiple-choice Each correct response count as “1” and each incorrect response count as “0.”	0–26
SexKen-ID. Sexual Knowledge, Experience, Feelings, and Needs of People with mild intellectual disabilities (McCabe, 1993; McCabe et al., 1999)	Sexual Knowledge, Experience, Feelings, and Needs	Assessing of Sexual Knowledge, Experience, Feelings, and Needs of people with mild intellectual disabilities	248	(Items in knowledge area): Friendship: 23 *(1);* Dating and intimacy: 16 *(2);* Marriage: 16 *(2);* Body part identification: 21 (*21*); Sex and sex education: 16 *(1);* Menstruation: 16 *(11);* Sexual interaction: 52 (*21); C*ontraception: 19 *(9);* Pregnancy, abortion, and childbirth: 24 *(15);* Sexually transmitted diseases: 19 *(11), M*asturbation: 16 (*3);* Homosexuality: 10 (*1)*	Each correct response counts as “1” or “2” for the dichotomous scale and Likert scale ranging from 1–5. The opened knowledge questions are scored 0, 1, or 2 depending on the accuracy of the responses	Friendship:*0–2;* Dating and intimacy: *0–4;* Marriage:*0–4;* Body part identification:* 0–42;* Sex and sex education*: 0–2;* Menstruation: *0–22;* Sexual interaction: *0–42;* Contraception: *0–18;* Pregnancy, abortion, and childbirth: *0–30;* Sexually transmitted diseases: *0–22;* Masturbation: *0–6;* Homosexuality: *0–2*
Assessment of Knowledge, Attitudes, Experiences, and Needs Questionnaire (Siebelink et al., 2006)	Sexual knowledge, sexual attitudes, sexual and relational experiences, and sexual and relational needs	Investigating the sexual knowledge, attitudes, experience, and needs of people with mild to moderate intellectual disabilities	28	Area Knowledge (Pregnancy, sexually transmitted disease, condoms): 4	Knowledge: three options choices (*“Yes,” “No,” or “I don’t know”*); and open questions	0–4
GSKQ. The General Sexual Knowledge Questionnaire (Talbot & Langdon, 2006)	Sexual Knowledge	Updating an outdated but existing assessment tool, the Bender Sexual Knowledge Questionnaire (BSKQ; Bender et al., 1983) for people with and without intellectual disability	63	Physiology A (Pictures): 16 and B (Questions): 13; Sexual intercourse: 10; Pregnancy: 8; Contraception: 5; Sexually transmitted diseases: 8; Sexuality: 3	1 point for each correct answer; however, they may score more than 1 point for each question, depending on the nature of the question	0–110

**Table 3:** Methodological qualities of the individual studies

Instrument	References	PROM development		Content validity			Structural validity	Internal consistency	Reliability	Construct validity	Responsiveness	
		PROM design	Pilot test	Relevance^a^	Comprehensibility^b^	Comprehensiveness^a^					Comparison between subgroups	Comparison before and after intervention
BSKQ	Bender et al. (1983)	I	I	NR	NR	NR	NR	NR	NR	NR	D	I
ASK	Galea et al. (2004)	I	I	A	I	A	NR	VG	A	NR	NR	NR
	Yueh-Ching et al. (2020)	NR	NR	NR	NR	NR	NR	VG	NR	NR	NR	D
DSARss	Gil-Llario et al. (2020)	I	I	D	I	D	A	VG	A	A	NR	NR
	Gil-Llario et al. (2023a)	NR	NR	NR	NR	NR	NR	VG	VG	NR	NR	VG
	Gil-Llario et al. (2022)	NR	NR	NR	NR	NR	NR	VG	NR	NR	D	NR
ISK-ID	Gil-Llario et al. (2021)	I	I	A	I	A	D	NR	NR	NR	NR	D
	Gil-Llario et al. (2023a)	NR	NR	NR	NR	NR	NR	VG	VG	NR	NR	A
	Gil-Llario et al. (2023b)	NR	NR	NR	NR	NR	NR	VG	NR	NR	NR	NR
Illustrated Scale Measuring the Sexual‐Abuse Prevention Knowledge of Female	Liou (2014)	I	I	A	I	A	A	VG	NR	NR	NR	NR
Pictorial Sexual Knowledge Scale for Male	Liou (2022)	I	I	A	I	A	A	VG	NR	NR	NR	NR
SexKen-ID	McCabe and Cummins (1996)	NR	NR	NR	NR	NR	NR	VG	A	NR	D	NR
	McCabe et al. (1994)	NR	NR	NR	NR	NR	NR	NR	NR	NR	D	NR
	McCabe et al. (1999)	D	D	D	D	D	NR	VG	A	NR	NR	NR
	Garwood and McCabe (2000)	NR	NR	NR	NR	NR	NR	VG	NR	NR	NR	I
	Murphy and O’Callaghan (2004)	NR	NR	NR	NR	NR	NR	NR	I	A	D	NR
Knowledge, Attitudes, Experiences, and Needs Questionnaire	Siebelink et al. (2006)	I	I	I	I	VG	NR	VG	NR	NR	NR	NR
GSKQ	Talbot and Langdon (2006)	I	I	D	I	D	NR	VG	NR	NR	D	NR
	Brkić-Jovanović et al. (2021)	NR	NR	NR	NR	NR	NR	VG	A	A	NR	NR

^a^Asking professionals; ^b^Asking people with intellectual disabilities. Ratings: A = Adequate, D = Doubtful, I = Inadequate, NR = No reported, VG = Very good

**Table 4:** Content validity

Instrument	Reference	Relevance		Comprehensibility		Comprehensiveness	
		PROM development study	Content validity study	Development study	Content validity	Development study	Content validity
BSKQ	Bender et al. (1983)	?	?	?	?	?	?
ASK	Galea et al. (2004)	?	±	?	?	?	+
	Yueh-Ching et al. (2020)	?	?	?	?	?	?
DSARss	Gil-Llario et al. (2020)	?	±	?	?	?	+
	Gil-Llario et al. (2023a)	?	?	?	?	?	?
	Gil-Llario et al. (2022)	?	?	?	?	?	?
ISK-ID	Gil-Llario et al. (2021)	?	±	?	?	?	+
	Gil-Llario et al. (2023a)	?	?	?	?	?	?
	Gil-Llario et al. (2023b)	?	?	?	?	?	?
Illustrated Scale Measuring the Sexual‐Abuse Prevention Knowledge of Female	Liou (2014)	?	±	?	?	?	+
Pictorial Sexual Knowledge Scale for Male	Liou (2022)	?	±	?	?	?	+
SexKen-ID	McCabe and Cummins (1996)	?	?	?	?	?	?
	McCabe et al. (1994)	?	?	?	?	?	?
	McCabe et al. (1999)	+	±	+	+	+	+
	Garwood and McCabe (2000)	?	?	?	?	?	?
	Murphy and O’Callaghan (2004)	?	?	?	?	?	?
Knowledge, Attitudes, Experiences, and Needs Questionnaire	Siebelink et al. (2006)	?	?	?	?	?	+
GSKQ	Talbot and Langdon (2006)	?	±	?	?	?	+
	Brkić-Jovanović et al. (2021)	?	?	?	?	?	?

The reviewers’ ratings were not involved

**Table 5:** Overall ratings of content validity of PROMS

Instrument (Reference)	Relevance		Comprehensibility		Comprehensiveness	
	Overall rating	Quality evidence	Overall rating	Quality evidence	Overall rating	Quality evidence
BSKQ	?	?	?	?	?	?
ASK	+	M	?	?	+	M
DSARss	+	L	?	?	+	L
ISK-ID	+	M	?	?	+	M
Illustrated Scale Measuring the Sexual‐Abuse Prevention Knowledge of Female	+	M	?	?	+	M
Pictorial Sexual Knowledge Scale for Male	+	M	?	?	+	M
SexKen-ID	+	L	+	L	+	L
Knowledge, Attitudes, Experiences, and Needs Questionnaire	+	VL	?	?	+	M
GSKQ	+	L	?	?	+	L

The quality of content validity (relevance, comprehensiveness, and comprehensibility) per study and content of the instrument was rated using the criteria for good content validity (Terwee et al., 2018); “+” sufficient; ? indeterminate (due to less robust psychometric data); “−” insufficient; ± inconsistent; NR: not reported psychometric data). The quality of evidence (confidence level for the overall quality rating of each psychometric property) was rated using a modified GRADE

Our aim was to analyze the psychometric properties of sexuality knowledge assessment tools because we only analyzed the measurement properties of the subscale for evaluating sexuality knowledge.

## Results

### Systematic Search

**Fig. 1:**
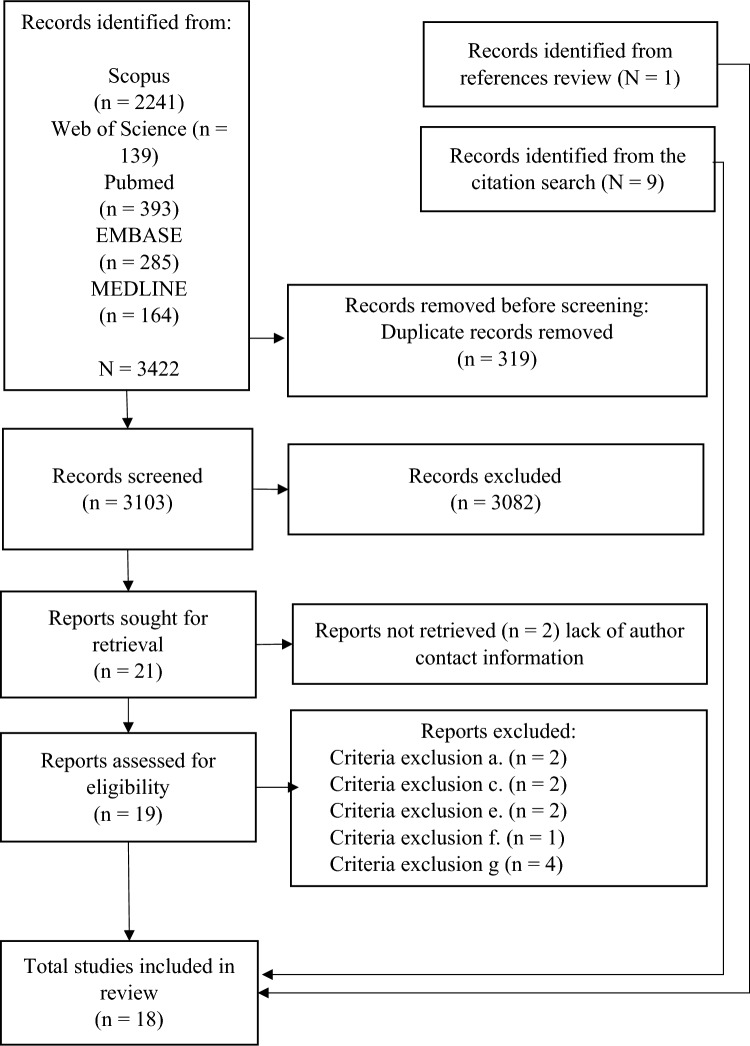
PRISMA flow diagram (Page et al., 2021)

The initial search revealed 3422 potentially eligible documents (Fig. 1). After removing the 319 duplicate studies, the titles and abstracts of the 3103 remaining studies were examined. A total of 3082 studies were excluded because they did not meet the inclusion criteria. Therefore, 21 articles were examined for full reading, and 8 studies whose references were reviewed were included; thus, we obtained one more study for analysis. To identify studies that used these instruments, a citation search was performed on Google Scholar backward and forward in time. Thus, nine studies were identified. The total number of studies included in this review was 18. The agreement between the reviewers for abstract selection was particularly strong, as evidenced by a weighted kappa (κ) of 0.85 (95% CI [0.820, 0.891]), indicating very good consistency. Similarly, the agreement for article selection remained robust, with a weighted kappa of 0.84 (95% CI [0.806, 0.876]). For coding, the inter-reviewer reliability was quantified with a kappa score of 0.78, which falls within a substantial range of agreement and suggests the consistent application of coding criteria across reviewers. Likewise, the quality assessment of the included studies demonstrated a high level of agreement with a kappa score of 0.81, confirming that the quality evaluation was uniformly rigorous and methodologically sound.

### Characteristics of the Included Studies and Instruments

The characteristics of the 18 included studies are presented in Appendix 2. The number of participants ranged between 33 and 345. The age ranged from 12 to 69 years. According to the level of intellectual disability, the sample was composed of participants with mild intellectual disability (Garwood & McCabe, 2000; Gil-Llario et al., 2021, 2022; McCabe & Cummins, 1996; McCabe et al., 1994, 1999), moderate intellectual disability (Brkić-Jovanović et al., 2021), mild and moderate intellectual disability (Galea et al., 2004; Gil-Llario et al., 2020; Siebelink et al., 2006; Talbot & Langdon, 2006), or mild, moderate, and severe intellectual disability (Liou, 2014, 2022; Yueh-Ching et al., 2020). Bender et al. (1983) and Gil-Llario et al. (2023b) did not report the level of intellectual disability of the participants. The participants with intellectual disability were delinquents in Bender et al. (1983) and Talbot and Langdon (2006), young students in Liou (2014, 2022), and adults who were clients of support network specialist disability services (Galea et al., 2004; Gil-Llario et al., 2020, 2021, 2022, 2023a, 2023b; McCabe et al., 1999; Murphy & O’Callaghan, 2004; Siebelink et al., 2006; Yueh-Ching et al., 2020). Studies were conducted in Australia (*n* = 5), Spain (*n* = 5), the UK (*n* = 3), Taiwan (*n* = 3), Serbia (*n* = 1), and the Netherlands (*n* = 1). Only one study reported the measurement properties of more than one tool of assessment (Gil-Llario et al., 2023a).

### Methodological Quality of the Included Studies

Eight of the studies that described PROM design were rated as inadequate quality because neither involved the target population. The findings of McCabe et al. (1999) were doubtful because the method used for the analysis of the data was not clear. A pilot test was only performed by McCabe et al. (1999) and was rated as doubtful because it was not clear if the patients were asked about comprehensibility. Eight studies asked people with intellectual disability or professionals about at least one of the three aspects of content validity (relevance, comprehensiveness, and comprehensibility). The methodological quality of studies on relevance was rated from doubtful to adequate, comprehensiveness was rated from doubtful to very good, and comprehensibility was scored as inadequate quality for all but one that ranged from doubtful (McCabe et al., 1999). Structural validity (*n* = 4), internal consistency (*n* = 15), cross-cultural validity/measurement invariance (*n* = 3), reliability (*n* = 8), construct validity (convergent validity) (*n* = 3), and responsiveness (*n* = 11) were assessed among the nine studies. Some studies measured more than one psychometric property, but no study assessed all the psychometric properties of the COSMIN. No information was retrieved on measurement error, cross-validity, or criterion validity in any study. The methodological quality of the studies on structural validity ranged from doubtful to adequate. The internal consistency was scored as very good. The quality of all studies on construct validity was scored as adequate, and reliability was scored as doubtful. Finally, the methodological quality of the responsiveness property ranged from inadequate to very good. These quality ratings were due to the preferred statistical analysis according to the COSMIN standards not being used.

### Psychometric Properties and Quality of Evidence of the Instruments

Ratings on PROM development and content validity were based on the scoring of content validity studies according to the 10 criteria for good content validity. Only McCabe et al. (1999) rated this development study as sufficient. According to the content validity study, seven studies were rated as inconsistent in relevance, and one study was scored as sufficient (McCabe et al., 1999). All content validity studies on comprehensibility were rated as indeterminate except for SexKen-ID. This is due to the study performed by McCabe et al. (1999), which was rated as sufficient. Finally, eight content validity studies on comprehensiveness were rated as sufficient. Other psychometric properties of the PROMs for each study are described in Appendix 3. Structural validity and internal consistency were rated as sufficient only in studies for DSARs and ISK-ID (Gil-Llario et al., 2020, 2021, 2023a, 2023b). This is because the other studies used less robust factor analysis (EFA) without data available to verify the applicability of the EFA (Watkins, 2018) or because structural validity was not disclosed. As a consequence, internal consistency was rated as insufficient because these studies did not meet the criterion of “at least low evidence for sufficient structural validity.” Only one study (Gil-Llario et al., 2023a) met the COSMIN criteria for good psychometric reliability because it used robust statistical methods, while the others used methods not recommended by the COSMIN guide (Spearman’s correlation coefficients and κ). This study revealed inconsistent results for the DSARs and insufficient results for the ISK-ID because the ICC was lower than 0.70. The construct validity was determined to be sufficient in three studies because the results supported this hypothesis (Brkić-Jovanović et al., 2021; Gil-Llario et al., 2020; Murphy & O’Callaghan, 2004). Responsiveness studies were scored as inconsistent in the works by Gil-Llario et al. (2023a) for DSARs and Gil-Llario et al. (2021) for ISK-ID because the effect size found for the intervention was not as expected. However, the study performed by Gil-Llario et al. (2023a) using the ISK-ID was rated as sufficient because the results were in line with this hypothesis.

All but one instrument (BSKQ) received overall ratings that were sufficient in relevance and comprehensiveness, with quality evidence that ranged from very low (knowledge, attitudes, experiences, and needs questionnaire) to moderate (ASK, ISK-ID, Illustrated Scale Measuring the Sexual-Abuse Prevention Knowledge of Female, and Pictorial Sexual Knowledge Scale for Male). All but SexKen-ID obtained indeterminate overall ratings of comprehensibility. Only SexKen-ID obtained an overall rating of sufficient comprehensibility with low-quality evidence.

As shown in Table 6, structural validity was sufficient, with moderate and low-quality evidence for two of the four instruments (DSARs and ISK-ID, respectively). The internal consistency was rated as sufficient for DSARs and ISK with low-quality evidence. For DSARs, the level of evidence was downgraded due to inconsistencies. The reliability of only these DSARs and ISK-ID instruments met the COSMIN criteria for good psychometric properties, but the DSARs were inconsistent, and the ISK-ID was rated as insufficient with high-quality evidence. The construct validity of the DSARs and the GSKQ was determined to be sufficient with high-quality evidence, and the SexKen-ID was rated as sufficient with moderate quality due to the sample size. Regarding responsiveness, DSARs were rated as inconsistent. The ISK-ID was rated as sufficient with moderate-quality evidence. The quality of the ISK-ID data was reduced because some of the results were inconsistent. BSKQ, ASK, SexKen-ID, and GSKQ were indeterminate since studies that did not report statistical data were preferred according to the COMIN criteria.

**Table 6:** Overall rating of the psychometric properties of PROMS and (quality of the evidence)

Instrument	Structural validity	Internal consistency	Reliability	Construct validity	Responsiveness	
					Comparison between subgroups	Comparison before and after intervention
BSKQ	NR	NR	NR	NR	?	?
ASK	NR	?	?	NR	NR	?
DSARss	+ (Moderate)	+ (Low)	?	+ (High)	±	±
ISK-ID	+ (Low)	+ (Low)	− (High)	NR	NR	+ (High)
Illustrated Scale Measuring the Sexual‐Abuse Prevention Knowledge of Female	?	?	NR	NR	NR	NR
Pictorial Sexual Knowledge Scale for Male	?	?	NR	NR	NR	NR
SexKen-ID	NR	?	?	+ (Moderate)	?	?
Knowledge, Attitudes, Experiences, and Needs Questionnaire	NR	?	NR	NR	NR	NR
GSKQ	NR	?	NR	+ (High)	?	NR

The overall quality of psychometric properties was rated using the criteria for good psychometric properties (Mokkink et al. 2018; Prinsen et al., 2018); + sufficient rating; ? indeterminate rating (due to less robust psychometric data); − insufficient rating; ± inconsistent rating; NR: not reported psychometric data); Data and ratings on each psychometric property per instrument are available in Supplementary Appendix 2. The quality of evidence (confidence level for the overall quality rating of each psychometric property) was rated using a modified GRADE approach (Mokkink et al. 2018; Prinsen et al., 2018): High: high level of confidence, Moderate: moderate level of confidence, Low: low level of confidence, Very Low: very low level of confidence, NR: not reported overall rating of psychometric properties

### Recommendation of PROMs

According to the recommendations of the COSMIN guide for formulating evidence‐based and fully transparent recommendations on the most suitable PROM for use in an evaluative application (Prinsen et al., 2018), the included PROMs were categorized into three categories: (A) PROMs with evidence for sufficient content validity (any level) and at least low-quality evidence for sufficient internal consistency; (B) PROMs categorized neither in A nor C; and (C) PROMs with high-quality evidence for insufficient measurement.

Thus, the DSARs and ISK-ID met the criteria for category A. These tools can be recommended for use, and the results obtained with these PROMs can be trusted, although more studies are needed to verify whether these tools are comprehensible for people with intellectual disability. The BSKQ, the ASK, the Illustrated Scale Measuring the Sexual Abuse Prevention Knowledge of Females, the Pictorial Sexual Knowledge Scale for Males, the SexKen-ID, the Knowledge, Attitudes, Experiences, and Needs Questionnaire, and the GSKQ have the potential to be recommended for use, but further research is needed to assess the quality of these PROMs (category B). In particular, the structural validity of the BSKQ, ASK, SexKen-ID, Knowledge, Attitudes, Experiences, and Needs Questionnaire, and GSKQ should be analyzed to meet the criteria of the COSMIN. The Illustrated Scale Measuring the Sexual Abuse Prevention Knowledge of Females and Pictorial Sexual Knowledge Scale for Males used EFA, but information about how the analysis was performed was not available; thus, the quality evidence of structural validity and internal consistency was not graded.

## Discussion

The purpose of this systematic review was to critically evaluate, compare, and summarize the measurement quality and psychometrical properties of all self-reported sexuality knowledge questionnaires for people with intellectual disability using the COSMIN guidelines. Eighteen studies on the psychometric properties of nine instruments were identified in this review. Most of the studies reported on two or fewer psychometric properties of the instrument, although the COSMIN taxonomy identifies nine measurement properties. These properties are classified into three domains: reliability, which includes internal consistency, reliability, and measurement error properties; validity, which includes content validity, structural validity, hypothesis testing for construct validity, cross‐cultural validity, and criterion validity; and responsiveness (Prinsen et al., 2018). Therefore, it is necessary to study these domains more completely.

In general, the methodological quality of individual studies for content validity, structural validity, and reliability was adequate and notably excellent for internal consistency. However, future analyses using the most preferred statistical methods (Prinsen et al., 2018) are needed for all the instruments, particularly concerning structural validity and reliability. Moreover, comprehensive studies on responsiveness, construct validity, cross-cultural validity, measurement error, and criterion validity are necessary to thoroughly assess the quality of the nine instruments under review.

It is crucial to highlight that individuals with intellectual disabilities were directly involved only in the development of the SexKen-ID scale. This involvement is significant because it ensures that the assessment tools are appropriately tailored to the actual comprehension levels and needs of the target population. Engaging individuals with intellectual disabilities in the development process not only enhances the relevance and effectiveness of the instruments but also respects and upholds their agency and input in research that impacts their lives. Given this successful involvement, it is recommended that future instrument development processes incorporate similar participatory approaches. Researchers might consider conducting pilot studies or structured interviews to further refine the items and response options, ensuring they are understandable and accurately measure what they intend to within this group.

Furthermore, most instruments were developed nearly 15 years ago and may not adequately reflect the current social and cultural contexts or the evolving understanding of sexuality and disability (Thompson et al., 2016). The necessity to update these tools cannot be overstated; it ensures that they remain relevant and sensitive to contemporary issues such as digital sexuality, consent, and the broadening spectrum of gender identities, which were not as prominently recognized or understood in the past as they are today. Instruments such as DSARs (Gil-Llario et al., 2020) and ISK-ID (Gil-Llario et al., 2021) have shown potential for ongoing use in research and in clinical practice. However, their effectiveness and reliability need to be validated across different linguistic and cultural groups, especially non-Spanish speaking populations, to ensure that they are universally applicable and effective.

Additionally, other tools, such as the BSKQ (Bender et al., 1983), the ASK (Butler et al., 2003), the Illustrated Scale Measuring the Sexual-Abuse Prevention Knowledge of Females (Liou, 2014), the Pictorial Sexual Knowledge Scale for Males (Liou, 2022), the SexKen-ID (McCabe, 1993), the Knowledge, Attitudes, Experiences, and Needs Questionnaire (Siebelink et al., 2006), and the GSKQ (Talbot & Langdon, 2006), also have potential for recommendation. Nevertheless, these instruments require thorough re-evaluation to validate their quality and applicability in current contexts. This re-evaluation should particularly focus on their structural validity, cross-cultural applicability, and ability to effectively measure the intended constructs in a way that is both inclusive and representative of the diversity within the population of people with intellectual disability.

The findings highlight a critical need for updated tools in sex education for individuals with intellectual disability. Effective sex education should be based on accurate and current assessment tools that reflect modern understanding and realities of sexual behavior and safety. Current instruments can help educators tailor content that is both comprehensible and relevant to this population, addressing specific educational gaps and enhancing overall sexual well-being.

Equally important is the development of self-protective skills through these educational programs. Accurate assessment tools will enable the identification of areas where individuals with intellectual disability may lack awareness or understanding, thereby guiding targeted interventions to foster skills such as consent, recognition of inappropriate behaviors, and ways to seek help. These skills are crucial for enhancing personal safety and empowering individuals to navigate social and intimate situations more effectively.

### Limitations

This review had several limitations that should be mentioned. First, we used “the worst score method” for evaluating the measurement properties, as recommended by the COSMIN guidelines guaranteeing that the standards were met. However, this could undereevaluate the results of the studies. Moreover, manuals, theses, and books were not included in this review because of the lack of finances and the limitations of time.

### Conclusions

Currently, there is no PROM for sexual knowledge with high-level evidence on all the psychometric properties. Variations in the psychometric properties among the different reported PROMs suggest a need for further studies. DSARs and the ISK-ID demonstrate potential for use in research and clinical practice, but their psychometric qualities for non-Spanish populations should be evaluated. Other tools, such as BSKQ and ASK, require further research to fully assess their quality. The few instruments identified could show a low interest in the sexual health of people with intellectual disability. More studies on high-quality sexual knowledge PROMs are needed in future.

## Supplementary Information

Below is the link to the electronic supplementary material.

Supplementary file1 (DOCX 56 KB)

## Data Availability

The data and material presented in this study are available on request from the corresponding author.

## References

[CR1] American Psychiatric Association. (2022). *Diagnostic and statistical manual of mental disorders* (5th ed., text rev.). American Psychiatric Publishing.

[CR2] Bender, M. P., Aitman, J. B., Biggs, S. J., & Haug, U. (1983). Initial findings concerning a sexual knowledge questionnaire. *Journal of the British Institute of Mental Handicap (APEX),**11*, 168–169. 10.1111/j.1468-3156.1983.tb00172.x

[CR3] Borawska-Charko, M., Rohleder, P., & Finlay, W. M. L. (2017). The sexual health knowledge of people with intellectual disabilities: A review. *Sexuality Research & Social Policy,**14*, 393–409. 10.1007/s13178-016-0267-4

[CR4] Brkić-Jovanović, N., Runjo, V., Tamaš, D., Slavković, S., & Milankov, V. (2021). Persons with intellectual disability: Sexual behaviour, knowledge and assertiveness. *Slovenian Journal of Public Health,**60*(2), 82–89. 10.2478/sjph-2021-0013 33822835 10.2478/sjph-2021-0013 PMC8015653

[CR5] Butler, J., Leighton, D., & Galea, J. (2003). *The assessment of sexual knowledge*. Centre for Developmental Disability Health Victoria.

[CR6] Chodan, W., Häßler, F., & Reis, O. (2017). A randomized controlled trial on the effectiveness of a sexual abuse prevention programme for girls with intellectual disabilities: Study protocol. *Translational Developmental Psychiatry,**5*(1), 1407192. 10.1080/20017022.2017.1407192

[CR7] Darragh, J., Reynolds, L., Ellison, C., & Bellon, M. (2017). Let’s talk about sex: How people with intellectual disability in Australia engage with online social media and intimate relationships. *Cyberpsychology Journal of Psychosocial Research on Cyberspace,**11*(1), Article article 9. 10.5817/CP2017-1-9

[CR8] Estruch-García, V., Cervigón-Carrasco, V., Fernández-García, O., Elipe-Miravet, M., & Gil-Llario, M. D. (2021). Metodología de los programas de educación afectivo-sexual para personas con diversidad funcional intelectual: una revisión sistemática [Methodology of the affective-sexual education programs for people with intellectual disabilities: A systematic review]. *International Journal of Developmental and Educational Psychology,**2*(2), 421–432. 10.17060/ijodaep.2021.n2.v2.2250

[CR9] Finlay, W. M. L., & Lyons, E. (2001). Methodological issues in interviewing and using self-report questionnaires with people with mental retardation. *Psychological Assessment,**13*(3), 319–335. 10.1037/1040-3590.13.3.319 11556269 10.1037//1040-3590.13.3.319

[CR10] Galea, J., Butler, J., Iacono, T., & Leighton, D. (2004). The assessment of sexual knowledge in people with intellectual disability. *Journal of Intellectual and Developmental Disability,**29*(4), 350–365. 10.1080/13668250400014517

[CR11] Garwood, M., & McCabe, M. P. (2000). Impact of sex education programs on sexual knowledge and feelings of men with a mild intellectual disability. *Education and Training in Mental Retardation and Developmental Disabilities,**35*(3), 269–283.

[CR12] Gil-Llario, M. D., Ballester-Arnal, R., Morelll-Mengual, V., Caballero-Gascón, L., & Castro-Calvo, J. (2020). Development and psychometric properties of the Detection of Sexual Abuse Risk Screening Scale (DSARss). *Sexual Abuse,**32*(7), 851–877. 10.1177/1079063219858061 10.1177/1079063219858061 31248341

[CR13] Gil-Llario, M. D., Castro-Calvo, J., Fernández-García, O., Elipe-Miravet, M., & Ballester-Arnal, R. (2021). Estimating sexual knowledge of people with mild intellectual disability through a valid and reliable assessment scale: The ISK-ID. *Journal of Applied Research in Intellectual Disabilities, 35*, 988–1000. 10.1111/jar.12909 34132002 10.1111/jar.12909

[CR14] Gil-Llario, M. D., Fernández-García, O., Huedo-Medina, T., Estruch-García, V., & Ballester-Arnal, R. (2023a). Analysis of the differential efficacy of the reduced version over the extended version of an affective-sexual education program for adults with intellectual disabilities. *Archives of Sexual Behavior,**52*, 135–147. 10.1007/s10508-022-02407-3 36169777 10.1007/s10508-022-02407-3 PMC9517966

[CR15] Gil-Llario, M. D., Fernández-García, O., Huedo-Medina, T., Nebot-García, J., & Ballester-Arnal, R. (2023b). A multilevel model to assess the effectiveness of an affective-sexual education program for people with intellectual disabilities: The influence of participants’ characteristics. *Sexuality Research & Social Policy,**20*, 1105–1123. 10.1007/s13178-022-00784-x

[CR16] Gil-Llario, M. D., Morell-Mengual, V., Fernández-García, O., Ruiz-Palomino, E., & Ballester-Arnal, R. (2022). Factors associated with condom use in vaginal intercourse among Spanish adults with intellectual disability: Proposal for an explanatory model. *Research in Developmental Disabilities, 121*. 10.1016/j.ridd.2021.104157 34971990 10.1016/j.ridd.2021.104157

[CR17] Grieveo, A., McLaren, S., & Lindsay, R. W. (2006). An evaluation of research and training resources for the sex education of people with moderate to severe learning disabilities. *British Journal of Learning Disabilities,**35*, 30–37. 10.1111/j.1468-3156.2006.00401.x

[CR18] Gutiérrez-Bermejo, B., Flores, N., Amor, P. J., & Jenaro, C. (2021). Evidence of an im-plemented training program in consensual and responsible sexual relations for people with intellectual disabilities. *International Journal of Environmental Research and Public Health,**18*, 2323. 10.3390/ijerph18052323 33652989 10.3390/ijerph18052323 PMC7967667

[CR19] Hartley, S. L., & MacLean, W. E., Jr. (2006). A review of the reliability and validity of Likert-type scales for people with intellectual disability. *Journal of Intellectual Disability Research,**50*(11), 813–827. 10.1111/j.1365-2788.2006.00844.x 16999781 10.1111/j.1365-2788.2006.00844.x

[CR20] Hayashi, M., Arakida, M., & Ohashi, K. (2011). The effectiveness of a sex education program facilitating social skills for people with intellectual disability in Japan. *Journal of Intellectual & Developmental Disability,**36*(1), 11–19. 10.3109/13668250.2010.549463 21284415 10.3109/13668250.2010.549463

[CR21] Jahoda, A., & Pownall, J. (2014). Sexual understanding, sources of information and social networks; The reports of young people with intellectual disabilities and their non-disabled peers. *Journal of Intellectual Disability Research,**58*(5), 430–441. 10.1111/jir.12040 23600407 10.1111/jir.12040

[CR22] Leutar, Z., & Mihoković, M. (2007). Level of knowledge about sexuality of people with mental disabilities. *Sexuality and Disability,**25*(3), 93–109. 10.1007/s11195-007-9046-8

[CR23] Liou, W. Y. (2014). An illustrated scale measuring the sexual-abuse prevention knowledge of female high school students with intellectual disabilities in Taiwan. *Sexuality and Disability,**32*, 135–151. 10.1007/s11195-013-9312-x

[CR24] Liou, W. Y. (2022). A pictorial sexual knowledge scale for male high school students with intellectual disabilities in Taiwan. *Sexuality and Disability,**40*, 623–649. 10.1007/s11195-022-09750-2

[CR25] Long, C. G., Krawczyk, K. M., & Kenworthy, N. E. (2011). Assessing the sexual knowledge of women in secure settings: The development of a new screening measure. *British Journal of Learning Disabilities,**41*, 51–65. 10.1111/j.1468-3156.2011.00722.x

[CR26] Malik, P. B., Ashton-Schaeffer, C., & Kleiber, D. A. (1991). Interviewing young adults with mental retardation: A seldom used research method. *Therapeutic Recreation Journal,**25*, 60–73.

[CR27] Martinello, E. (2014). Reviewing strategies for risk reduction of sexual abuse of children with intellectual disabilities: A focus on early intervention. *Sexuality and Disability,**32*, 167–174. 10.1007/s11195-014-9345-9

[CR28] McCabe, M. P. (1993). *Sexual knowledge, experience and needs scale (SexKen)* (3rd ed.). Psychology Research Centre, Deakin University.

[CR29] McCabe, M. P., & Cummins, R. A. (1996). The sexual knowledge, experience, feelings and needs of people with mild intellectual disability. *Education and Training in Mental Retardation and Developmental Disabilities,**31*(1), 13–21.

[CR30] McCabe, M. P., Cummins, R. A., & Deeks, A. A. (1999). Construction and psychometric properties of sexuality scales: Sex knowledge, experience, and needs scales for people with intellectual disabilities (SexKen-ID), people with physical disabilities (SexKen-PD), and the general population (SexKen-GP). *Research in Developmental Disabilities,**20*(4), 241–254. 10.1016/S0891-4222(99)00007-4 10425653 10.1016/s0891-4222(99)00007-4

[CR31] McCabe, M. P., Cummins, R. A., & Reid, S. B. (1994). An empirical study of the sexual abuse of people with intellectual disability. *Sexuality and Disability,**12*(4), 297–306. 10.1007/BF02575321

[CR32] Mitra, M., Mouradian, V. E., Fox, M. H., & Pratt, C. (2016). Prevalence and characteristics of sexual violence against men with disabilities. *American Journal of Preventive Medicine,**50*(3), 311–317. 10.1016/j.amepre.2015.07.030 26474667 10.1016/j.amepre.2015.07.030 PMC4762736

[CR33] Mokkink, L. B., de Vet, H. C. W., Prinsen, C. A. C., Patrick, D. L., Alonso, J., Bouler, L. M., & Terwee, C. B. (2018). COSMIN risk of bias checklist for systematic reviews of patient-reported outcome measures. *Quality of Life Research,**27*, 1171–1179. 10.1007/s11136-017-1765-4 29260445 10.1007/s11136-017-1765-4 PMC5891552

[CR34] Murphy, G. H., & O’Callaghan, A. (2004). Capacity of adults with intellectual disabilities to consent to sexual relationships. *Psychological Medicine,**34*, 1347–1357. 10.1017/S0033291704001941 15697061 10.1017/s0033291704001941

[CR35] Navarro, Y., Torrico, E., & López, M. J. (2010). Programa de intervención psicosexual en personas con discapacidad intelectual [Psychosexual intervention program in people with intellectual disability]. *Educación y Diversidad,**4*(2), 75–92.

[CR36] Page, M. J., McKenzie, J. E., Bossuyt, P. M., Boutron, I., Hoffmann, T. C., Mulrow, C. D., Shamseer, L., Tetzlaff, J. M., Akl, E. A., Brennan, S. E., Chou, R., Glanville, J., Grimshaw, J. M., Hróbjartsson, A., Lalu, M. M., Li, T., Loder, E. W., Mayo-Wilson, E., McDonald, S., … Moher, D. (2021). The PRISMA 2020 statement: an updated guideline for reporting systematic reviews. *BMJ,**372*, n71. 10.1136/bmj.n71 33782057 10.1136/bmj.n71 PMC8005924

[CR37] Prinsen, C. A. C., Mokkink, L. B., Bouter, L. M., Alonso, J., Patrick, D. L., de Vet, H. C. W., & Terwee, C. B. (2018). COSMIN guideline for systematic reviews of patient-reported outcome measures. *Quality of Life Research,**27*, 1147–1157. 10.1007/s11136-018-1798-3 29435801 10.1007/s11136-018-1798-3 PMC5891568

[CR38] Schaafsma, D., Kok, G., Stoffelen, J. M. T., & Curfs, L. M. G. (2017). People with intellectual disabilities talk about sexuality: Implications for the development of sex education. *Sexuality and Disability,**35*, 21–38. 10.1007/s11195-016-9466-4 28250541 10.1007/s11195-016-9466-4 PMC5306299

[CR39] Siebelink, E. M., de Joong, M. D. T., Taal, E., & Roelvink, L. (2006). Sexuality and people with intellectual disabilities: Assessment of knowledge attitudes, experiences, and needs. *Mental Retardation,**44*(4), 283–294. 10.1352/0047-6765(2006)44[283:sapwid]2.0.co;2 16834465 10.1352/0047-6765(2006)44[283:SAPWID]2.0.CO;2

[CR40] Sigelman, C. K., Winer, J. L., & Schoenrock, C. J. (1982). The responsiveness of mentally retarded persons to questions. *Education & Training of the Mentally Retarded,**17*(2), 120–124.

[CR41] Talbot, T. J., & Langdon, P. E. (2006). A revised sexual knowledge assessment tool for people with intellectual disabilities: Is sexual knowledge related to sexual offending behaviour. *Journal of Intellectual Disability Research,**50*(7), 523–531. 10.1111/j.1365-2788.2006.00801.x 16774637 10.1111/j.1365-2788.2006.00801.x

[CR42] Terwee, C. B., Prinsen, C. A. C., Chiarotto, A., Westerman, M. J., Patrick, D. L., Alonso, J., Bouter, L. M., de Vet, H. C. W., & Mokkink, L. B. (2018). COSMIN methodology for evaluating the content validity of patient-reported outcome measures: A Delphi study. *Quality of Life Research,**27*(5), 1159–1170. 10.1007/s11136-018-1829-0 29550964 10.1007/s11136-018-1829-0 PMC5891557

[CR43] Thompson, V. R., Stancliffe, R. J., Wolson, N. J., & Broom, A. (2016). The content, usefulness and usability of sexual knowledge assessment tools for people with intellectual disability. *Sexuality and Disability,**34*, 495–512. 10.1007/s11195-016-9458-4

[CR44] Tomsa, R., Gutu, S., Cojocaru, D., Gutiérrez-Bermejo, B., Flores, N., & Jenaro, C. (2021). Prevalence of sexual abuse in adults with intellectual disability: Systematic review and meta-analysis. *International Journal of Environmental Research and Public Health,**18*(4), Article 1980. 10.3390/ijerph18041980 33670753 10.3390/ijerph18041980 PMC7921934

[CR45] van den Toren, S. J., de Haas, S., Dalmijn, E., Feenstra, H., & van Berlo, W. (2021). A mixed methods evaluation of Girls’ Talk+: A sexuality education programme for girls with mild intellectual disabilities. *Journal of Applied Research in Intellectual Disabilities,**34*(6), 1462–1471. 10.1111/jar.12933 10.1111/jar.12933 34414637

[CR46] Vizcaino, L., & Aciego, R. (2015). Assessment of emotional-sexual education experience for people with intellectual disabilities. *Siglo Cero,**46*(4), 45–58. 10.14201/scero2015464455

[CR47] Watkins, M. W. (2018). Exploratory factor analysis: A guide to best practice. *Journal of Black Psychology,**44*(3), 219–246. 10.1177/0095798418771807

[CR48] World Health Organization. (2006). *Defining sexual health: Report of a technical consultation on sexual health* [January 28, 31, 2022]. Author.

[CR49] Yueh-Ching, C., Zxy-Yann, J. L., Bo-Wei, C., & Chwen-Jen, L. (2020). Awareness of sexual rights and empowerment: Quantitative and qualitative evaluation of a sexual health intervention for adults with intellectual disability. *Journal of Sex Research,**57*(9), 1202–1216. 10.1080/00224499.2019.1629383 31276427 10.1080/00224499.2019.1629383

