# Supplementary material for: COSMIN Review About Assessment Tools for Sexuality Knowledge in People with Intellectual Disability
Source: Arch Sex Behav. 2025 Nov 24;54(10):4023–38. doi: 10.1007/s10508-025-03260-w (PMC12753555; doi:10.1007/s10508-025-03260-w)
Supplement: Supplementary file 1 — Supplementary file1 (DOCX 56 KB) [file 10508_2025_3260_MOESM1_ESM.docx]

***Appendix 1. Search strategy***

| **Data base** | **Keywords** | **N studies found** |
| --- | --- | --- |
| **Web of Science** | ('knowledge' OR 'understanding') AND ('sexuality' OR 'sexual health' OR 'sexual abuse' OR 'sexual relationships' OR 'violence' OR 'gender identities' OR 'gender roles' OR 'anatomy' OR 'intimate parts' OR 'sexual orientation' OR 'eroticism' OR 'pleasure' OR 'intimacy' OR 'reproduction' OR 'sexually transmitted diseases' OR ' sexual risk') AND ('intellectual disability' OR 'disorder intellectual development' OR 'intellectual functional diversity' OR 'mental retardation') AND ('measure*' OR 'assess*' OR 'tool' OR 'instrument' OR ' scale' OR ' question*' OR ' battery' OR ' inventory' OR ' index' OR 'form' OR 'evaluation') AND (construction OR psychometric properties OR develop* OR validity OR validation OR reliability OR responsiveness OR interpretability) (Topic) | 139 |
| **Pubmed** | ('knowledge' OR 'understanding') AND ('sexuality' OR 'sexual health' OR 'sexual abuse' OR 'sexual relationships' OR 'violence' OR 'gender identities' OR 'gender roles' OR 'anatomy' OR 'intimate parts' OR 'sexual orientation' OR 'eroticism' OR 'pleasure' OR 'intimacy' OR 'reproduction' OR 'sexually transmitted diseases' OR ' sexual risk') AND ('intellectual disability' OR 'disorder intellectual development' OR 'intellectual functional diversity' OR 'mental retardation') AND ('measure*' OR 'assess*' OR 'tool' OR 'instrument' OR ' scale' OR ' question*' OR ' battery' OR ' inventory' OR ' index' OR 'form' OR 'evaluation') AND (construction OR psychometric properties OR develop* OR validity OR validation OR reliability OR responsiveness OR interpretability) | 593 |
| **Embase** | ('knowledge' OR 'understanding') AND ('sex*' OR 'sexual health' OR 'sexual abuse' OR 'sexual relationships' OR 'violence' OR 'gender identities' OR 'gender roles' OR 'anatomy' OR 'intimate parts' OR 'sexual orientation' OR 'eroticism' OR 'pleasure' OR 'intimacy' OR 'reproduction' OR 'sexually transmitted diseases' OR 'sex* risk* behavior*') AND ('intellectual disability' OR 'disorder intellectual development' OR 'intellectual functional diversity' OR 'mental retardation') AND ('measure*' OR 'assess*' OR 'tool' OR 'instrument' OR 'scale' OR 'question*' OR 'battery' OR 'inventory' OR 'index' OR 'form' OR 'evaluation') AND ((construction OR psychometric) AND properties OR develop* OR valid* OR reliability OR responsiveness OR interpretability) | 285 |
| **Medline** | ('knowledge' OR 'understanding') AND ('sex*' OR 'sexual health' OR 'sexual abuse' OR 'sexual relationships' OR 'violence' OR 'gender identities' OR 'gender roles' OR 'anatomy' OR 'intimate parts' OR 'sexual orientation' OR 'eroticism' OR 'pleasure' OR 'intimacy' OR 'reproduction' OR 'sexually transmitted diseases' OR 'sex* risk* behavior*') AND ('intellectual disability' OR 'disorder intellectual development' OR 'intellectual functional diversity' OR 'mental retardation') AND ('measure*' OR 'assess*' OR 'tool' OR 'instrument' OR 'scale' OR 'question*' OR 'battery' OR 'inventory' OR 'index' OR 'form' OR 'evaluation') AND ((construction OR psychometric) AND properties OR develop* OR valid* OR reliability OR responsiveness OR interpretability) | 164 |
| **Scopus** | ALL ( "intellectual disability" OR "disorder intellectual development" OR "intellectual functional diversity" OR "mental retardation" ) AND ( knowledge OR understanding ) AND ( sexuality OR "sexual health" OR "sexual abuse" OR "sexual relationships" OR violence OR "gender identities" OR "gender roles" OR anatomy OR "intimate parts" OR "sexual orientation" OR eroticism OR pleasure OR intimacy OR reproduction OR "sexually transmitted diseases" OR "sexual risk behavior" ) AND ( measure OR measurement OR assessment OR assess OR tool OR instrument OR scale OR question* OR battery OR inventory OR index OR form OR evaluation ) AND ( construction OR psychometric AND properties OR development OR develop OR validity OR validation OR reliability OR responsiveness OR interpretability ) AND ( LIMIT-TO ( DOCTYPE , "ar" ) ) AND ( LIMIT-TO ( SUBJAREA , "PSYC" ) OR LIMIT-TO ( SUBJAREA , "MEDI" ) OR LIMIT-TO ( SUBJAREA , "SOCI" ) OR LIMIT-TO ( SUBJAREA , "HEAL" ) OR LIMIT-TO ( SUBJAREA , "NURS" ) ) | 2241 |

**Appendix 2. Characteristics of the studies included.**

| **Prom**  **(Ref)** | **Study ref** | **N** | **Age**  **Mean (Sd) Range** | **Gender**  **% (n) Female** | **Disease Severity**  **%** | **Setting** | **Country**  **(Language**) | **Response Rate** |
| --- | --- | --- | --- | --- | --- | --- | --- | --- |
| **BSK**  **(Bender et al., 1983)** | Bender et al. (1983) | G_1_= 15 | 16.0 | 0% | Delinquents “hardcore” | N/R | UK  (English) | 93.75% |
|  |  | G_2_= 18 | 24.1 | 0% | Mentally handicapped young adults some with psychiatric difficulties | N/R |  | 100% |
| **ASK**  **(Butler et al., 2003)** | Galea et al. (2004) | 96 | 31.5  18-57 | 46.75%  (42) | 75% people with mild ID  and 22% moderate ID | Specialist disability supporting | Australia  Victoria | NR |
|  | Yueh-Chin et al. (2020) | GE_1_= 33 | 27.1  (6.5)  19-41 | 30.3% (10) | 60.6 % people with mild/moderate ID, and 39.4% with severe ID | Specialist disability service | Taiwan | 89.47% |
|  |  | GC_2_= 11 | 30.7 (8.2)  21-43 | 45.5%  (5) | 90.9% people with mild/moderate ID, and 9.1% with severe ID |  |  |  |
|  |  | GC_3_= 7 | 30.7  (6.2)  23-41 | 0 | 57.1 % people with mild/moderate ID, and 42.9% with severe ID |  |  |  |
|  |  | GE_4_= 19 | 31.7  (5.9)  21-42 | 68.4%  (13) | 73.7 % people with mild/moderate ID, and 26.3 % with severe ID |  |  |  |
|  |  | GE_5_= 17 | 28.7  (6.5)  20-43 | 82.4%  (14) | 100% people with severe ID |  |  |  |
| **DSAR**  **(Gil-Llario et al., 2020)** | Gil-Llario et al. (2020) | 246 | 37.8  (10.4)  19-64 | 55.3% (136) | People with mild or moderate ID  45.12% (n=111) suffered from other mental and/or physical conditions: (ADHD; 4.5%, n = 5), motor sensory disorders (4.5%, n = 5), hearing loss or deafness (2.7%, n = 3), epilepsy (2.7%, n = 3), and schizophrenia (2.7%, n = 3). | Specialist disability service | Spain,  Valencia  (Spanish) | NR |
|  | Gil-Llario et al. (2023a) | 208 | 37.23 (10.66)  19-67 | 50.48%  (105) | People with ID | Specialist disability services | Spain,  Valencia  (Spanish) | NR |
|  | Gil-Llario et al. (2022) | 253 | 38.52  (10.48) | 43.9%  (111) | People with mild ID | Specialist disability services | Spain,  Valencia  (Spanish) | NR |
| **ISK-ID**  **(Gil-Llario et al., 2020)** | Gil-Llario et al. (2021) | 345 | 37.77  (10.50)  19-69 | 44.9%  (155) | People with mild ID | Specialist disability services | Spain,  Valencia  (Spanish) | 100% |
|  | Gil-Llario et al. (2023a) | 208 | 37.23 (10.66)  19-67 | 50.48%  (105) | People with ID | Specialist disability services | Spain,  Valencia  (Spanish) | NR |
|  | Gil-Llario et al. (2023b) | 254 | 37.25  (10.59)  19-67 | 53% (135) | People with ID | Specialist disability services | Spain,  Valencia  (Spanish) | NR |
| **Illustrated Scale Measuring the Sexual‐Abuse Prevention Knowledge of Female**  **(Liou 2014)** | Liou (2014) | 196 | 16. 5 (10.60)  14.11 - 18 | 100% | 36 (18.4%) mild,  141 (71.9%) moderate and 19 (9.7%) severe | High school students with intellectual disabilities | Taiwan | 90.32% |
| **Pictorial Sexual Knowledge Scale for Male**  **(Liou, 2022)** | Liou (2022) | 181 | 17.2 (11.70)  15.7-20. 7 | 0% | 21% (n=38) with mild, 67.4% (n=122) moderate, and 11.6% (n=21) severe ID | Disabilities studying at special education schools in Taiwan | Taiwan | 91.87% |
| **SexKen-ID**  **McCabe (1993)**  (McCabe et al., 1999) | McCabe & Cummins (1996) | G_1_= 30 | 25.2  16-40 | 10%  (2) | People with mild ID | People living independently in the community | Victoria, Australia  (English) | NR |
|  |  | G_2_= 50 | 20.6  17-35 | 36%  (18) | People without ID | Students |  |  |
|  | McCabe et al. (1994) | G_1_= 30 | 25.2  16-40 | 40%  (12) | People with mild ID | People living independently in the community | Victoria, Australia  (English) | NR |
|  |  | G_2_= 50 | 20.6  17-35 | 36%  (18) | People without ID | Students |  |  |
|  | McCabe et al. (1999) | G_1_= 67 | 27.62 (4.81) | 47.26%  (32) | People with mild ID | Specialist disability service  People living independently in the community | Victoria, Australia  (English) | NR |
|  |  | G_2_=60 | 28.65  (5.23) | 45%  (27) | People with congenital physical disabilities |  |  |  |
|  |  | G_3_=100 | 30.10  (5.67) | 60%  (60) | People without ID | General population |  |  |
|  | Garwood & McCabe (2000) | G_1_= 3 | 19  (6.5)  12-25 | 0% | People with mild ID | Living with their parents | Victoria, Australia  (English) | 85.71% |
|  |  | G_2_=3 | 31.6  (3.22)  18-32 | 0% |  |  |  |  |
|  | Murphy & O’Callaghan  (2004) | G_1_= 60 | 37.6 (10.4) | 50%  (30) | People with ID (IQ = 59. 8 (S.D.= 8. 9) | Specialist disability service | Kent and South London | 98% |
|  |  | G_2_=60 | 16.6 (0.55) | 50%  (30) | Young people | Wide-ability schools |  |  |
| **Assessment of Knowledge, Attitudes, Experiences, and Needs Questionnaire**  **(Siebelink et al., 2006)** | Siebelink et al. (2006) | 76 | 18-30: 18  30-50: 40  >50: 18 | 38%  (29) | Mild and moderate ID | Specialist disability service | Netherlands  (English) | NR |
| **GSKQ**  **(Talbot & Langdon, 2006)** | Talbot & Langdon (2006) | G_1_=12 | 35.32 (13.42) | 0% | Sex offenders with ID and a history of engagement in treatment  Mild and moderate ID (IQ: 64.9; SD=6.79) | Secure learning disabilities services | UK, East Anglia  (English) | NR |
|  |  | G_2_=13 |  | 0% |  | Recruited from residential units |  |  |
|  |  | G_3_=28 | 33.52 (9.80) | 17.87 % (5) | Non-offenders with an intellectual disability  Mild and moderate ID (IQ: 62.4; SD=6.43) |  |  |  |
|  |  | G_4_=10 | 37.1  (15.3) | 50%  (5) | People without ID | Administrative and support staff working within services for people with IDs, |  |  |
|  | Brkić-Jovanović et al. (2021) | 100 | 47.64 (9.66)  23-63 | 54%  (54) | People with moderate ID | Residing in institutional housing | Serbia  (Serbian) | 100% |

***Appendix 3: Rating of the psychometric properties of individual studies.***

| **Psychometric Property** | **Instrument** | **Reference** | **Sample Size** | **Results** | **Ratings*** |
| --- | --- | --- | --- | --- | --- |
| **Structural validity** | **DSARss** | Gil-Llario et al. (2020) | 246 | EFA: 5-factor structure. Data reported verifying the applicability of the EFA: Kaiser–Meyer–Olkin (KMO) index (KMO = 0.720), Barlett’s test of sphericity, χ^2^(171) = 915.6, p < 0.001, and the determinant of the polychoric correlation matrix (0.0269) were calculated. | + |
|  | **ISK-ID** | Gil-Llario et al. (2021) | 345 | Computed the “unidimTest” function from the “ltm” package. This test confirmed that the six dimensions of the ISK-ID were unidimensional (*p* > 0.05). To determine whether the 6 unidimensional domains of the ISK-ID measured a common underlying factor general sexual knowledge. Mplus software was used. The scree plot showed that there were six factors with eigenvalues above 1.  2PL method to analyse our data. Item parameters were analysed using the “mirt” package. *a* value (between 0.016 – 30.67).  5 of the 34 items on the ISK-ID obtained negative *b* values.  28 of the 34 items on the ISK-ID obtained a negative MDIFF value. Three items (items 15, 26 and 34) were below −3. Six items obtained positive MDIFF values (between 0.02 and 1.17),  23 of the 34 items on the ISK-ID had appropriate discriminant power, whereas 11 items had very high discrimination power (MDISC between 3.44 and 10.71).  *Theta* value for the global test ability (0.0035). The highest *theta* value (0.0112) was reached in the ‘Homosexuality’ domain, whereas the lowest value corresponded to the ‘Body Image and sexual communication’ domain (−0.0061), knowledge about the ‘concept of sexuality’ (−0.0037); by contrast, they had good knowledge in terms of information about sexual practices (‘Sexual practices’), information about healthy sexual relationships (‘Sexual health’), and awareness of how to interact with their partner in a relationship (‘Dating, intimacy, and sexual assertiveness’) | + |
|  | **Illustrated Scale Measuring the Sexual‐Abuse Prevention Knowledge of Female** | Liou (2014) | 196 | EFA: 5-factor structure  Data not reported to verify the applicability of the EFA | ? |
|  | **Pictorial Sexual Knowledge Scale for Male** | Liou (2022) | 181 | EFA: 6-factor structure  Data not reported to verify the applicability of the EFA | ? |
| **Internal consistency** | **ASK**  **Knowledge section an QKQ** | Galea et al. (2004) | 96 | No evidence of structural validity. Scales and KR 20 = 0.89 | ? |
|  |  | Yueh-Ching et al. (2019) |  | QKQ: No evidence of structural validity. Scales and Cronbach’s Alpha = 0.70 | ? |
|  | **DSARss** | Gil-Llario et al. (2020) | 246 | The criteria for “At least low evidence for sufficient structural validity” was met and Cronbach’s Alpha ranged between 0.70 and 0.93 | + |
|  |  | Gil-Llario et al. (2023a) | 208 | The criteria for “At least low evidence for sufficient structural validity” was met and Cronbach’s Alpha ranged between 0.50 and 0.71. Total Scale Cronbach’s Alpha=.51. | - |
|  |  | Gil-Llario et al. (2022) | 253 | The criteria for “At least low evidence for sufficient structural validity” was met and Omega coefficient ranged between 0.74 and 0.93. | + |
|  | **ISK-ID** | Gil-Llario et al. (2023a) | 208 | The criteria for “At least low evidence for sufficient structural validity” was met and Cronbach’s Alpha ranged between 0.54 and 0.67. Total Scale Cronbach’s Alpha= 0.76 | + |
|  |  | Gil-Llario et al. (2023b) | 254 | The criteria for “At least low evidence for sufficient structural validity” was met and Cronbach’s Alpha ranged between 0.51 and 0.70. Total Scale Cronbach’s Alpha= 0.79 | + |
|  | **Illustrated Scale Measuring the Sexual‐Abuse Prevention Knowledge of Female** | Liou (2014) | 196 | The criteria for “At least low evidence for sufficient structural validity” was not met, and of the 5 subscales of true/false items, Cronbach’s Alpha ranged from 0.54 to 0.92; of the 5 subscales for the multiple-choice items Cronbach’s Alpha ranged from 0.51 to 0.65. | ? |
|  | **Pictorial Sexual Knowledge Scale for Male** | Liou (2022) | 181 | The criteria for “At least low evidence for sufficient structural validity” was not met. 6 subscales Cronbach’s Alpha ranged from 0.58 to 0.78. | ? |
|  | **SexKen-ID** | McCabe et al. (1999) | G_1_=67 | No evidence of structural validity. Cronbach’s Alpha between 0.47 and 0.96 | ? |
|  |  | Garwood and McCabe (2000) | 6 | No evidence of structural validity. Cronbach’s Alpha between 0.47 and 0.86 (not reported Cronbach’s Alpha of all areas that composed the test | ? |
|  | **Knowledge, Attitudes, Experiences, and Needs Questionnaire** | Siebelink et al. (2006) | 76 | No evidence of structural validity. Cronbach’s Alpha = 0.69 | ? |
|  | **GSKQ** | Talbot & Langdon (2006) | 63 | No evidence of structural validity. Cronbach’s Alpha between 0.35 and 0.94 | ? |
|  |  | Brkić-Jovanović et al. (2021) | 100 | No evidence of structural validity. Cronbach’s Alpha = 0.80 | ? |
| **Reliability** | **ASK**  **Knowledge section** | Galea et al. (2004) | 33 | Inter-Rater. Correlation between section totals for scores by each rater (Spearman’s rho) indicated a range of 0.83 to 0.99. | ? |
|  |  |  | 96 | Test-retest correlations were between 0.62 and 1. | ? |
|  | **DSARss** | Gil-Llario et al. (2020) | 246 | Test-retest correlation 6 months after the first administration was 0.47 for the total score and from 0.20 to 0.52 for the subscales (r factor 1 = 0.52, r factor 2 = 0.40, r factor 3 = 0.32, r factor 4 = 0.20). All the correlations were significant at p < 0.001. | ? |
|  |  | Gil-Llario et al. (2023a) | 208 | ICC ranged pre-test (0.02 - 0.78)  ICC ranged post-test (0.12 - 0.78) | +/- |
|  | **ISK-ID** | Gil-Llario et al. (2023a) | 208 | ICC ranged pre-test (0.07 - 0.23)  ICC ranged post-test (0.06 - 0.43) | - |
|  | **SexKen-ID** | McCabe et al., (1999) | 30 | Test-Retest correlation coefficients were between 0.23 and 0.96 with p-values ranging between < 0.05 to < 0.001. | ? |
|  |  | Murphy & O’Callaghan (2004) | G_1_= 60  G_2_=60 | Inter-Rater. K no reported. The mean percentage agreements for the Understanding Consent and Abuse measure and the Vignettes measure were 93.4% and 89.3% respectively (ranges 87–100% and 81–94%). | ? |
|  | **GSKQ** | Brkić-Jovanović et al. (2021) | 100 | Test-retest (r = 0.86) | ? |
| **Construct validity** | **DSARss** | Gil-Llario et al. (2020) | 246 | AF-5 (Self-concept–5): academic and work, family, emotional, and social self-concept did not correlate with any DSARss factors. The psychic scale correlated with “Factor 3. Risk factors and self-protection skills” (r = -0.17).  INICO-FEAPS (quality-of-life): Self-determination, personal growth and social relationships did not significative correlation with DSARss factors. The rights scale correlated with Factor 1 (Acceptance of the abuse due to affection) r = -0.17.  Correlations with (changes in) instruments measuring unrelated constructs should be < 0.30.  Inappropriate sexual and partner behaviours:  “*If you like a person, you can force him or her to kiss you*” correlated with “factor 2. Denial of the risk associated with places “(r = 0.13), factor 3 (r = 0.31), “factor 4. “Lack of awareness of intimacy rules” (r = 0.13).  *“If you like a person, you can force him or her to go hand in hand*” correlated with (r = 0.49) and the factors: f1 (r = 0.28), f2 (r = 0.18), f3 (r = 0.38), f4 (r = 0.47).  *“Have you ever touched your privates in public?”) as well as with general sexual behaviours* (e.g., vaginal, or oral sex)” correlated with the total score (r = 0.49) and the factors: f1 (r = 0.28), f2 (r = 0.18), f3 (r = 0.38), f4 (r = 0.47).  Hypothesis: Correlations with (changes in) instruments measuring related, but dissimilar constructs should be lower, e.g., 0.30 ‐ 0.50. | + (H3+) |
|  | **SexKen-ID** | Murphy & O’Callaghan (2004) | G_1_= 60 | SexKen-ID (all areas) and only the Homosexuality area correlated significantly with Understanding Consent and Abuse measure (rho= 0.42 and rho= 0.60, respectively).  Hypothesis: Correlations with (changes in) instruments measuring related, but dissimilar constructs should be lower, e.g., 0.30 ‐ 0.50. | + |
|  | **GSKQ** | Brkić-Jovanović et al. (2021) | 100 | GSKQ correlated (r= 0.360) with the What if test (instruments that evaluate sexual-abuse prevention)  Hypothesis: Correlations with (changes in) instruments measuring related, but dissimilar constructs should be lower, e.g., 0.30 ‐ 0.50. | + |
| **Responsiveness** |  | | | | |
| **10c. Construct approach: (e.g., hypotheses testing comparison between subgroups)** | **BSK** | Bender et al. (1983) | G_1_= 35  G_2_=18 | Cohen’s d not reported | ? |
|  | **DSARss** | Gil-Llario et al. (2022) | 253 | Significant in the factor of lack of awareness of intimacy rules (t(80) = 2.730; p = 0.008, d Cohen= 0.60) but not in the factors of acceptance of the abuse due to affection, denial of the risk associated with places, and risk factors and self-protection skills. | +/-  (H1+/3-) |
|  |  |  |  |  |  |
|  | **SexKen-ID** | McCabe & Cummins (1996) | G_1_= 30  G_2_=50 | Cohen’s d not reported | ? |
|  |  | McCabe et al. (1994) | G_1_= 30  G_2_=50 | Cohen’s d not reported | ? |
|  |  | McCabe et al. (1999) | G_1_= 67  G_2_=60  G_3_=100 | Cohen’s d not reported | ? |
|  |  | Murphy & O’Callaghan (2004) | G_1_= 60  G_2_=60 | Cohen’s d not reported | ? |
|  | **GSKQ** | Talbot & Langdon (2006) | G_1_= 12  G_2_=13  G_3_= 28  G_4_=10 | Cohen’s d not reported | ? |
| **10d. Construct approach: (e.g., hypotheses testing before and after intervention)** | **BSKQ** | Bender et al. (1983) | GE_1_= 15  GE_2_=18 | Cohen’s d not reported | ? |
|  | **ASK**  **QKQ** | Yueh-Ching et al. (2019) | GE_1_= 33  GC_2_=11  GC_3_=7  GE_4_= 19  GE_5_=17 | Cohen’s d not reported | ? |
|  | **DSARss** | Gil-Llario, Fernández-García, Huedo-Medina, Estruch-García, & Ballester-Arnal  (2023) | 208 | Not Effect was found for factor 1 (d= 0.04) and factor 3 (d= 0.03)  Factor 2 obtained a small effect (d= 0.28)  Factor 4 obtained intermated effect (d= 0.60)  Hypothesis: small and intermediate effect (d= 0.20 - 0.70) is expected because the SRE Program have 16 sessions of 1 hour and people have ID. | +/-  (H2+/H2-) |
|  | **ISK-ID** | Gil-Llario et al. (2021) | 345 | Mean Scores in ‘Body image and sexual communication’, ‘Sexual practices’, ‘Homosexuality’, and ‘Dating, intimacy, and sexual assertiveness’ significantly increased after the implementation of the educational intervention with a small effect (d between 0.25 and 0.34). Scores in factor 1 (‘Concept of sexuality’) also increased after the implementation of the program, but the differences did not reach statistical significance (d = 0.14). Finally, the level of knowledge after the intervention decreased slightly in one domain: ‘sexual health’.  Hypothesis: small and intermediate effect (d= 0.20 - 0.70) is expected because the SRE Program have 16 sessions of 1 hour and people have ID. | +/-  (H4+/2-) |
|  |  | Gil-Llario, Fernández-García, Huedo-Medina, Estruch-García, & Ballester-Arnal  (2023) | 208 | Effect sizes comparing the extended and reduced version was low for four factors and the total scale (total scale: d= 0.29, factor 1: d= 0.27, factor 3: d= -0.21, factor 5: d= -0.35, factor 6: d= 0.35), intermediate for Body image and sexual communication’ (d= 0.58), and without effect for homosexuality factor (d= 0.03)  hypothesis: small and intermediate effect (d= 0.20 - 0.70) is expected because the SRE Program have 16 sessions of 1 hour and people have ID. | + (H6+/1-) |
|  | **SexKen-ID** | Garwood and McCabe (2000) | GE_1_= 33  GE_2_=11 | Cohen’s d not reported | ? |
|  |  | Murphy & O’Callaghan (2004) | G_1_= 60  G_2_=60 | Cohen’s d not reported | ? |

***** + sufficient rating;? indeterminate rating (due to less robust psychometric data); - insufficient rating; +/- inconsistent rating; NR: not reported psychometric data); GC= Control Group, GE= Experimental group
